# Ginkgolide as a Promising Multi-Target Therapeutic for Alzheimer's Disease: Targeting ApoE4 and Beyond

**DOI:** 10.2174/0113816128386836250723134001

**Published:** 2025-07-31

**Authors:** Saba Beigh, Mansoor Alsahag, Ali Alisaac, Sajad Ahmad Dar, Mohammad H. Alyami, Soma E. Ajlan, Hesham A. Malak, Abdullateef Abdullah Alshehri

**Affiliations:** 1 Department of Public Health, Faculty of Applied Medical Sciences, Al-Baha University, Al-Baha, Saudi Arabia;; 2 Faculty of Applied Medical Sciences, Al-Baha University, Al-Baha, Saudi Arabia;; 3 Department of Nursing, College of Nursing and Health Sciences, Jazan University, Jazan, 45142, Saudi Arabia;; 4 Department of Pharmaceutics, College of Pharmacy, Najran University, Najran, 66462, Saudi Arabia;; 5 Medical Microbiology and Immunology Department, Faculty of Medicine, Menoufia University, Shibin Al Kawm, Egypt;; 6 Department of Biology, Faculty of Science, Umm Al-Qura University, Makkah, Saudi Arabia;; 7 Department of Clinical Laboratory Sciences, College of Applied Medical Sciences, Najran University, P.O. Box 1988, Najran, Saudi Arabia

**Keywords:** Ginkgolide, Alzheimer's disease, apolipoprotein E4 (ApoE4), molecular docking, blood-brain barrier, multi-target therapy

## Abstract

**Introduction:**

The progressive neurodegenerative disease known as Alzheimer's disease (AD) is typified by neuroinflammation, amyloid-beta buildup, and cognitive impairment. Current pharmacological treatments merely alleviate symptoms, despite extensive research, which underscores the need for innovative, multi-target medicines. Since apolipoprotein E4 (ApoE4) is a significant genetic risk factor linked to the development of AD, it is a potentially effective treatment target. With their neuroprotective qualities, natural substances like Ginkgolide may help treat some diseases. This study investigates Ginkgolide's potential as a multi-target treatment for AD, with a particular emphasis on how it interacts with the ApoE4 N-terminal domain.

**Methods:**

The interaction between Ginkgolide and ApoE4 (PDB ID: 8AX8) was assessed using pharmacokinetic profiling, molecular docking, and molecular dynamics (MD) simulations. MD simulations were used to determine stability, and AutoDock Vina was used to obtain the binding affinity. To predict pharmacokinetics and toxicity, SwissADME and PkCSM were employed. The effectiveness of ginkgolide was contextualized using comparative docking with curcumin and resveratrol.

**Results:**

Ginkgolide formed sustained hydrophobic contacts with important sites and demonstrated a substantial binding affinity (-7.1 kcal/mol) to ApoE4. MD simulations verified negligible fluctuations and complex stability over 100 ns. Pharmacokinetics showed no significant toxicity risks, good gastrointestinal absorption, and favorable blood-brain barrier permeability. In terms of binding affinity and stability, ginkgolide fared better than curcumin and resveratrol, indicating its greater therapeutic potential.

**Discussion:**

The results indicate that ginkgolide effectively binds and stabilizes the ApoE4 N-terminal domain, supporting its potential role in modulating a key pathological factor in Alzheimer’s disease. Its superior pharmacokinetic profile and interaction dynamics compared to curcumin and resveratrol suggest a broader therapeutic relevance. These *in silico* insights provide a mechanistic basis for further investigation into ginkgolide’s neuroprotective effects.

**Conclusion:**

The results demonstrated ginkgolide as a potentially effective multi-target treatment for AD through ApoE4 regulation. It is a better option than other natural chemicals because of its potent binding affinity, stability, and pharmacokinetics. These findings highlight the value of *in silico* methods in the early stages of drug discovery and the need for additional experimental support before they can be used in clinical settings.

## 1 INTRODUCTION

Alzheimer’s disease (AD) can be described as an unbearable neurodegenerative condition that is the most common cause of dementia, affecting millions of people around the world. This progressive illness is explained by deterioration in cognitive skills such as thinking and reasoning, loss of memory, and alteration of behavior, which results in dependency and a heavy burden on the will of the caregivers and the healthcare systems [1]. It is estimated that because of the wider population aging, the occurrence of births of AD will increase greatly, which puts a lot of emphasis on finding effective means of treatment for this illness.

The occurrence of AD is caused due to molecular and cellular events working in alarming unison [2]. At its basis, the AD is characterized by two key components: an extracellular accumulation of plaques (Aβ) and an intracellular aggregation of hyperphosphorylated tau protein that forms neurofibrillary tangles. These changes in AD also result in altered synaptic function and stability of the neural circuits with time and apoptosis of the neurons [3]. The pattern of AD, which is characterized by the loss of the ability to create new memory, logically fits that the damage starts with the region of the brain that is responsible for the function - the hippocampus, and also the ‘thinking’ part of the brain - the cerebral cortex [4]. Plaques and tangles apart, AD is coupled with microglial and astrocytic dysfunction that amplifies the neuronal assault by inflammation. Oxidative stress and mitochondrial dysfunction further enhance cellular toxicity, creating a vicious cycle of neurodegeneration [5]. These processes underscore the multifactorial nature of AD, pointing to the need for therapies that address multiple pathological targets simultaneously. Genetics also plays a very important role in determining an individual's susceptibility to AD [6].

The genetic heterogeneity observed in late-onset Alzheimer's disease (LOAD) presents significant challenges when it comes to pinpointing the specific genes or pathways directly responsible for triggering the onset of clinical pathology [7]. Despite these challenges, one gene has consistently emerged as a key player in the pathogenesis of LOAD: apolipoprotein E (APOE) [7]. This gene is widely regarded as the most influential genetic risk factor for the disease, and its impact on numerous pathogenic pathways contributing to Alzheimer’s disease is well-documented. In humans, three major APOE alleles exist: ε2 (APOE2), ε3 (APOE3), and ε4 (APOE4) [8]. Growing evidence suggests that the APOE4 gene increases the risk of developing Alzheimer's disease by both introducing harmful effects and reducing protective functions. On the other hand, APOE2 has been found to offer some protection against AD-related damage, though the exact mechanisms behind this remain unclear. Interestingly, Insel and colleagues reported that carrying an APOE2 gene along with APOE4 might provide some protection against amyloid-beta (Aβ) buildup compared to having APOE3 [8]. However, other studies have shown that individuals with both APOE2 and APOE4 have a risk profile more similar to those with APOE4 alone, suggesting that the increased risk linked to APOE4 outweighs the potential protection from APOE2 [9]. These alleles are associated with varying levels of risk for developing Alzheimer's disease. Of these, the ε4 allele is the most strongly linked to an increased risk of developing LOAD, and the presence of this allele significantly accelerates the disease process. Studies have shown that individuals carrying the ε4 allele have a higher susceptibility to cognitive decline and the accumulation of neurotoxic proteins, making them more likely to develop the disease [10]. In contrast, the ε2 allele is thought to have a protective effect against AD, although its precise mechanism remains under active investigation [10]. The various isoforms of APOE specifically, the structural differences between the ε2, ε3, and ε4 isoforms, have significant implications for the ability of APOE to interact with key molecules involved in Alzheimer’s disease, such as lipids, receptors, and amyloid-β (Aβ). Aβ is a protein that accumulates in plaques in the brain, a hallmark feature of Alzheimer's disease. These structural variations in the APOE isoforms result in different functional properties, which influence the progression of the disease [11].

Research in both human and animal models has shown that APOE isoforms play distinct and critical roles in modulating several pathological processes, including neuroinflammation, tau protein hyperphosphorylation, and the aggregation and clearance of amyloid-β [12]. These processes are central to the neurodegenerative nature of AD, and understanding how different APOE isoforms influence these pathways could provide valuable insights into disease progression and potential therapeutic targets. One of the key functions of APOE is its role in maintaining lipid homeostasis. Lipids such as cholesterol and triglycerides are essential for cellular function and structure, yet they are hydrophobic molecules that cannot dissolve in water. As a result, they must be transported throughout the body by lipoproteins—particles that are capable of interacting with both water and fat [13]. ApoE plays a crucial role in the transport of these lipids between various tissues and cell types, including the brain. This process is essential for the proper functioning of the central nervous system (CNS), as lipids are needed for the synthesis of neuronal membranes, myelin, and other critical components of brain cells [14]. In the peripheral circulation, the transport of lipids *via* lipoproteins is well-understood, with lipoproteins acting as carriers to shuttle lipids between the small intestine, liver, and other tissues. However, within the CNS, lipoproteins are often referred to as high-density lipoprotein (HDL)-like particles. While much is known about the role of lipoproteins in lipid transport, the precise characteristics of these HDL-like particles—such as their size, shape, and distribution within the brain—are still not fully understood. APOE plays an essential role in facilitating lipid transport between the peripheral circulation and the CNS, and it is believed that disruptions in this process may contribute to the onset and progression of Alzheimer's disease [15].

Beyond its role in lipid transport, APOE is also involved in regulating the integrity of the blood-brain barrier (BBB). The blood-brain barrier is a highly selective barrier that controls the flow of substances between the bloodstream and the brain [16]. ApoE, especially the ε4 isoform, is thought to influence the integrity of the BBB, potentially compromising its function and allowing harmful substances, such as amyloid-β, to accumulate in the brain. The ε4 allele has been shown to modulate amyloid-β metabolism and clearance, creating a favorable environment for amyloid plaques to form and contribute to neurodegeneration [17]. Additionally, ApoE4’s influence on lipid transport and synaptic plasticity can further exacerbate the neurotoxic environment, leading to neuronal damage and cognitive decline. In contrast, the APOE2 isoform has been found to possess protective effects against Alzheimer's disease [18]. These protective effects are thought to arise from the unique structural properties of ApoE2, which may promote the efficient clearance of amyloid-β and reduce neuroinflammation. The N-terminal domain of ApoE2, in particular, has garnered significant attention in recent years [18]. This domain is believed to play a critical role in modulating the functional properties of APOE and may provide insights into novel therapeutic strategies. Understanding the structural and functional subtleties of the APOE isoforms, particularly the differences between ApoE2 and ApoE4, is a major focus of current Alzheimer’s disease research. The potential therapeutic implications of APOE research are vast. Targeting APOE, particularly the ApoE4 isoform, offers an exciting opportunity to modify the trajectory of Alzheimer's disease and potentially prevent or delay its onset. Therapies that aim to modulate APOE's function, improve lipid transport, or enhance amyloid-β clearance could be key to developing effective treatments for Alzheimer's disease. Furthermore, understanding the protective role of ApoE2 may lead to novel strategies that enhance its beneficial effects, providing new avenues for intervention [19, 20].

In addition to genetic factors, environmental factors such as lifestyle, diet, and toxin exposure further modulate the risk for AD. Hypertension, diabetes, and obesity are chronic conditions associated with increased susceptibility, suggesting that neurological outcomes are influenced by systemic health [21]. These insights support the holistic view in the prevention and treatment of AD. The pharmacological options for AD remain limited, offering symptomatic benefits. Acetylcholinesterase inhibitors represent the mainstays of current pharmacological therapy, together with NMDA receptor antagonists such as memantine [22]. These symptomatic medications momentarily improve cognitive manifestations but do nothing to modify disease processes. Moreover, a series of failures in clinical trials targeting amyloid-beta and tau proteins underlines the challenges in the development of disease-modifying therapies. These failures outline the need to explore other approaches, including targeting upstream mechanisms and leveraging multi-targeted strategies. Furthermore, one big obstacle is the blood-brain barrier, which minimizes the amount of therapeutic agent that reaches the central nervous system. Overcoming these challenges will be important to advance the treatment of AD [23].

The natural compounds have evoked tremendous interest as therapeutic agents for AD. Products from various biological sources show a wide array of pharmacological activities: antioxidant, anti-inflammatory, and neuroprotective effects [24]. The structural diversity and bioavailability are other added advantages that make them attractive candidates for drug development. Among natural compounds, diterpene lactones represent Ginkgolide from Ginkgo biloba, was promising in preclinical studies [25]. Ginkgolide displays anti-amyloidogenic properties, modulates synaptic plasticity, and is protective against oxidative stress- all positioning it as a potential therapeutic agent for AD. The study of such compounds agrees with a recent emphasis on multi-targeted approaches against the complex pathology of the disease. Advances in computational methodologies have revolutionized drug discovery by enabling the identification and optimization of therapeutic candidates with unprecedented efficiency. Molecular docking, molecular dynamics simulations, and pharmacokinetics modeling provide a deep view of the drug-target interactions. These *in silico* methods have been used in conjunction with traditional experimental methods to reduce the time and cost associated with drug development [26]. Pharmacokinetics and toxicity assessments are an integral part of assessing the clinical potential of natural compounds. Tools such as ADMET analysis, which predict the properties including absorption, distribution, metabolism, excretion, and toxicity, ensure that the criteria of drug-likeness are possessed by the candidate molecules. Further clarification of binding affinities and interaction profiles by molecular docking studies and molecular dynamics simulations assesses stability and behavior under physiological conditions of drug-target complexes [27]. Thus, targeting ApoE4 is one of the encouraging approaches in the therapy of AD. ApoE4, being an important player in amyloid-beta metabolism and synaptic integrity, may have its pathogenic effects mitigated by functional modulation [28]. Structural studies have identified specific binding sites on ApoE4 that are amenable to therapeutic intervention, thus providing a foundation for drug design. Ginkgolide, with its favorable pharmacological profile, emerges as a compelling candidate for ApoE4 targeting [29]. Preclinical studies suggest that Ginkgolide can cross the blood-brain barrier, interact with key residues on ApoE4, and exert neuroprotective effects. Further support of this comes from various computational analyses that provided strong binding affinities and stable interactions with ApoE4 [30]. These findings point out the synergy between natural product research and computational drug discovery.

The integration of natural compounds into AD therapy is one paradigm shift, addressing key points including the value of multi-targeted approaches in holistic treatment strategies. This allows the researcher to accelerate the identification and optimization of therapeutic agents through computation, closing the gap between preclinical study and clinical application [31]. In this respect, experimental validation of the computational predictions would be required in further studies, especially in cell and animal models. Neuroprotection mechanisms exerted by compounds like Ginkgolide will be of high importance in clinical development for such drugs. Furthermore, combination therapies targeting different targets may significantly improve therapeutic efficacy and overcome the heterogeneity of AD [32]. The burden of AD calls for creative means of its prevention and management. Natural products, insights into genetics combined with modern computation-these bring new hope. Key player target strategies for therapeutic intervention targeting the players identified, such as ApoE4, and research into something like Ginkgolide, for which the path was opened here, can achieve transformative gains. Despite significant research on APOE's involvement in lipid metabolism and amyloid clearance, there is a critical gap in understanding the specific molecular pathways through which different APOE isoforms contribute to AD progression. Recent research suggests that multi-targeting natural compounds may offer superior therapeutic benefits. However, the potential of Ginkgolide, a bioactive terpenoid from Ginkgo biloba, remains largely unexplored for AD. This study provides a novel computational evaluation of Ginkgolide’s interactions with the N-terminal domain of Apolipoprotein E4 (ApoE4), a critical genetic risk factor for AD. Unlike previous studies on curcumin and resveratrol, our investigation uniquely demonstrates Ginkgolide’s superior pharmacokinetic properties, blood-brain barrier permeability, and binding affinity to ApoE4, underscoring its promise as a novel multi-target therapeutic agent. These findings contribute to the growing evidence supporting the use of computational approaches in early-stage drug discovery for neurodegenerative diseases.

## 2 MATERIALS AND METHODS

### 2.1 Structural Retrieval and Preparation

Key molecular targets associated with neurodegenerative disorders, particularly Alzheimer's disease, were identified from an extensive literature review. The selected protein structures included tau protein (PDB ID: 5O3T), amyloid beta-peptide (PDB ID: 5OQV), and apolipoprotein E (PDB ID: 8AX8). The three-dimensional structures of human apolipoprotein E isoforms (APOE2 and APOE3) were also retrieved PDB IDs 2L7B (NMR structure of full-length APOE3) and 1LE2 (X-ray diffraction structure of APOE2). These three-dimensional protein structures were retrieved from the Protein Data Bank (PDB) (https://www.rcsb.org/). The natural compounds selected for evaluation were obtained from PubChem (https://pubchem.ncbi.nlm.nih.gov/) in Structure Data Format (SDF) [33]. For positive control, Imipramine CID 3696 was selected for comparative analysis. To ensure structural accuracy and suitability for computational analysis, proteins were subjected to an optimization process. MODELLER v10.4 [34] (University of California, San Francisco, USA) was used to refine and reconstruct any missing residues or loops within the protein structures. PyMOL v2.5 [35] (Schrödinger, LLC, New York, USA) and Swiss PDB Viewer v4.1 [36] (Swiss Institute of Bioinformatics, Lausanne, Switzerland) were employed for structure visualization, validation, and correction of potential steric clashes or side-chain anomalies. The optimization process included energy minimization to improve structural stability, eliminate steric hindrances, and refine atomic positions. Additionally, all protein structures underwent pre-processing steps to remove non-essential molecules such as bound ligands, crystallographic water molecules, and extraneous ions that could interfere with docking analysis. These modifications ensured the accuracy and reliability of protein-ligand interactions for molecular docking and subsequent simulations.

### 2.2 Pharmacokinetics and Toxicity Analysis

The pharmacokinetic and toxicity profiles of the selected natural compounds were thoroughly assessed through a combination of advanced *in silico* predictive models. These models allowed for the evaluation of the drug-likeness and the absorption, distribution, metabolism, and excretion (ADME) properties of the compounds. To assess the ADME properties, SwissADME (Swiss Institute of Bioinformatics, Lausanne, Switzerland) and PkCSM (University of Cambridge, UK) was utilized, which provided insights into various pharmacokinetic parameters, including molecular weight (MW), lipophilicity (Log P), hydrogen bond donors and acceptors, number of rotatable bonds, gastrointestinal (GI) absorption, blood-brain barrier (BBB) permeability, and interactions with cytochrome P450 enzymes [36, 37]. In addition to the pharmacokinetic properties, toxicity profiles were evaluated using DataWarrior v5.5.0 (Actelion Pharmaceuticals Ltd., Allschwil, Switzerland). The *in-silico* toxicity screening assessed critical parameters such as acute and chronic toxicity, carcinogenicity, hepatotoxicity, cytotoxicity, mutagenicity, and genotoxicity risks [38]. The combination of SwissADME, PkCSM, and DataWarrior provided a comprehensive assessment of the potential drug candidates' pharmacokinetic feasibility and toxicity risk, enabling the selection of the most promising natural compounds for further molecular docking and dynamics studies.

### 2.3 Molecular Docking

Molecular docking analysis was performed to predict the binding affinities and interaction patterns of the selected natural compounds with the target proteins using AutoDock Vina v1.2.0 [39] (The Scripps Research Institute, La Jolla, CA, USA). The protein structures were energy-minimized and converted into PDBQT format using AutoDockTools v1.5.7, while the ligand structures were optimized with Avogadro v1.2.0 before also being converted into PDBQT format. A docking grid was generated centered on the active site of each target protein based on previously reported binding sites, with grid dimensions set to ensure sufficient space for ligand flexibility. The ligands were docked using AutoDock Vina’s genetic algorithm with a default exhaustiveness setting of 8, and the top 10 binding poses for each ligand were ranked according to their binding energy (in kcal/mol). Post-docking analysis was conducted using Chimera v1.15 [40], PyMOL v2.5 [41], and BIOVIA Discovery Studio Visualizer v21.1.0 [42] to visualize and analyze interactions such as hydrogen bonds, hydrophobic interactions, and π-π stacking interactions between the ligands and target proteins. The top-ranking compounds, based on binding energy, were then selected for further molecular dynamics (MD) simulations, ensuring the identification of the most promising natural compounds with strong binding affinities and favorable interaction profiles for potential therapeutic applications.

### 2.4 Molecular Dynamics Simulation

To assess the stability and conformational dynamics of the top-selected protein-ligand complexes, molecular dynamics (MD) simulations were conducted using Desmond v6.8 (Schrödinger, LLC, New York, USA) [43]. Each protein-ligand complex was placed in an explicit TIP3P water model within an orthorhombic box (10 × 10 × 10 Å) to ensure a fully solvated environment, with 0.15 M NaCl added to neutralize the system and mimic physiological ionic strength. The OPLS_2005 force field [44, 45] was applied for accurate parameterization of atomic interactions, and the system was minimized and equilibrated using the NPT ensemble (300 K, 1 atm pressure) to achieve a thermodynamically stable state. The production phase was run for 100 ns, with a 2 fs integration time step to capture real-time molecular fluctuations.

Molecular dynamics (MD) simulations are a computational technique used to study the movement of atoms and molecules over time. In the context of drug-protein interactions, MD simulations help to predict how a drug molecule (ligand) interacts with its target protein. These simulations reveal the stability and dynamics of such interactions by simulating the behavior of both the ligand and protein in a solvated environment, providing insights into how tightly the drug binds to the protein, whether any conformational changes occur, and the flexibility of the binding site. MD simulations thus offer a dynamic view of the interactions, which is crucial for understanding the potential efficacy of drug candidates. The simulation trajectories were analyzed to determine Root Mean Square Deviation (RMSD), evaluating overall stability of the complex, Root Mean Square Fluctuation (RMSF), measuring flexibility of specific residues, hydrogen bond analysis to monitor ligand-protein interactions over simulation time, and Radius of gyration (Rg) to determine compactness and structural integrity. The MD simulations provided valuable insights into ligand stability, dynamic behavior, and interaction strength, enabling the identification of potential drug candidates with strong and stable binding affinities.

### 2.5 Statistical and Computational Tools

All computational analyses were carried out on a high-performance computing workstation equipped with advanced specifications to ensure optimal performance and efficiency. The system was powered by an Intel Xeon Gold 6258R processor, featuring 24 cores running at 3.0 GHz, which provided the necessary computational power to handle large-scale simulations and complex calculations. For enhanced graphical processing and parallel computation, the workstation was equipped with an NVIDIA RTX 3090 GPU, boasting 24GB of VRAM, ensuring smooth execution of computational tasks, particularly those requiring intensive graphic rendering or machine learning processes. The workstation was also outfitted with 128 GB DDR4 RAM, offering ample memory capacity to support memory-intensive operations and simulations, minimizing the chances of bottlenecks and ensuring a seamless workflow. The operating system used was Ubuntu 22.04 LTS, a robust and reliable platform that supports a wide range of scientific and computational tools, further enhancing the efficiency and stability of the system during long and demanding computational analyses. This high-performance setup enabled the smooth execution of molecular docking, dynamics simulations, and toxicity assessments, ensuring that all the required analyses were conducted efficiently and accurately. Software Packages: Schrödinger Suite, AutoDock, Chimera, PyMOL, SwissADME, PkCSM, and DataWarrior

## 3 RESULTS

### 3.1 Structural Retrieval and Preparation

The structure of ApoE4 N-terminal domain, having PDB ID: 8AX8 was retrieved from RCSB Protein Databank (PDB). The structure of the protein was determined through the X-RAY Diffraction method, having a resolution of 1.55 Å, and is classified as a lipid-binding protein. It performs a variety of functions, including acting as a mediator of lipid transfer between organs and interstitial fluids. ApoE4 was chosen as the focus for this study due to its central role in Alzheimer's disease pathogenesis, particularly in amyloid-beta metabolism and synaptic plasticity, making it a critical target for therapeutic intervention. Targeting ApoE4 specifically could offer a promising avenue for therapeutic strategies due to its critical involvement in neurological disorders, especially Alzheimer’s disease, where it is implicated in the modulation of amyloid-beta deposition and neuroinflammation. Compared to other targets, ApoE4 presents a unique challenge and opportunity as its isoform-specific interactions with lipids and amyloid-beta could influence disease progression more directly. The 3D structures of ligands were retrieved from the PubChem database in SDF format. Fig. (**1**) represents the structure of the N-terminal domain of Apolipoprotein E4 (ApoE4) (PDB ID: 8AX8) along with its pharmacokinetics and toxicity analysis.

### 3.2 Pharmacokinetics and Toxicity Analysis

The ADMET analysis was performed by utilizing open-source tools including Swiss-ADME, PkCSM (https://biosig.lab.uq.edu.au/pkcsm), and Data Worrier. The properties under consideration were molecular weight, hydrogen bond donor, hydrogen bond acceptor, rotatable bonds, human intestinal absorption, blood-brain barrier permeability, CaCo2 permeability, water solubility, hepatotoxicity, AMES toxicity, carcinogenicity, mutagenicity, tumorigenicity, reproductive effects, irritant, oral rat acute toxicity, oral rat chronic toxicity, drug likeness, and Lipinski rule of five violation. Table **1** presents the predicted pharmacokinetic properties of the analyzed compounds.

### 3.3 Molecular Docking

Molecular docking was performed to determine the binding affinity, binding pose, and the interaction between two molecules [16, 17]. ADMET analysis was performed for a large number of compounds that were supposed to be effective for the treatment. Molecular docking of thirty compounds was performed, and it was observed that the binding affinity of Ginkgolide (CID_9909368) was -7.1 kcal/mol, which was the top one among them. Table **2** provides the compound IDs, names, 3D structures, and their respective binding energies. When comparing the results of the compounds tested to Imipramine (CID_3696), which you used as the control, it is observed that Imipramine exhibits docking scores of -6.3 kcal/mol for APOE2, -6.6 kcal/mol for APOE3, and -5.5 kcal/mol for APOE4. Compared to Imipramine, some compounds show stronger binding affinities across the APOE isoforms. Ginkgolide (CID_9909368), for example, binds more strongly to APOE2 with a score of -7.6 kcal/mol and exhibits strong binding to APOE3 and APOE4 with scores of -7.2 kcal/mol and -7.1 kcal/mol, respectively. Biacalein (CID_5281605) also demonstrates stronger binding to APOE2 (-7.0 kcal/mol) and APOE3 (-6.9 kcal/mol) than Imipramine. However, Imipramine shows the weakest binding to APOE4 when compared to all other compounds, including Salvianolic acid (CID_5281793) (-6.4 kcal/mol) and Epigallocatechin gallate (CID_65064) (-6.5 kcal/mol), which have slightly stronger interactions with APOE4. Therefore, while Imipramine remains a strong control, other compounds like Ginkgolide and Biacalein demonstrate potentially better binding profiles, especially for APOE2 and APOE3, and could be considered for further investigation (Table **2**).

The interactive residues were observed by using UCSF Chimera and Discovery Studio, and PyMOL software. On the basis of ADMET analysis and molecular docking, it was observed that Ginkgolide (CID_9909368) can become a potential drug to treat Alzheimer’s disease by specifically targeting ApoE4. Given ApoE4’s association with Alzheimer’s pathology, its inhibition might reduce amyloid-beta deposition, an essential factor in disease progression. Fig. (**2**) illustrates the complex of the Apolipoprotein E4 (ApoE4) N-terminal domain with Ginkgolide, while Fig. (**3**) highlights the molecular interactions between the ApoE4 N-terminal domain and Ginkgolide.

### 3.4 Molecular Dynamics Simulation

The graphical analysis of molecular dynamics simulation results reveals information about fluctuation, deviation, and equilibrium shown by biomolecules in a real-time environment. The stability of protein in interaction with specific small compounds was observed with the aid of molecular dynamics simulation. After pharmacokinetics analysis and molecular docking, the molecular dynamics simulation was performed on the selected 8AX8-Ginkgolide complex and also the control Imipramine. Root mean square deviation, root mean square fluctuation, protein-ligand contacts, and secondary structure analysis were determined by analysing trajectories.

The RMSD of both the protein and ligand for the Apoe4 complex with control Imipramine over time. The protein RMSD fluctuates between 1.2 Å and 5.4 Å, with several notable spikes, especially in the initial 20 nanoseconds. These significant fluctuations indicate possible structural rearrangements or instabilities in the Apoe4 protein upon ligand binding. The overall trend suggests that the protein undergoes relatively large conformational changes during the simulation.

The RMSD values for the Apoe4 complex with Ginkgolide, show a distinct pattern compared to the Imipramine condition. The protein RMSD is notably lower, fluctuating between 1.5 Å and 2.8 Å. This more stable behaviour indicates that Ginkgolide induces less structural variability in the protein compared to Imipramine, suggesting a more stable protein-ligand interaction throughout the simulation. However, there are still some fluctuations observed, particularly between 30 and 60 nanoseconds, which could reflect dynamic changes in protein-ligand interactions. The data from Figs. (**4A** and **4B**) provide insights into the dynamic behavior of the Apoe4 protein-ligand complex under the influence of two different ligands. In Fig. (**4A**), the protein bound with Imipramine exhibits larger RMSD fluctuations, indicating that the protein undergoes more structural rearrangements or instability, which may affect the stability of the overall complex. Conversely, in Fig. (**4B**), Ginkgolide leads to a more stable protein conformation, as evidenced by the smaller and less frequent RMSD fluctuations. This suggests that Ginkgolide might induce a more stable interaction between the protein and ligand. This stability, especially in the ligand position, suggests that Ginkgolide may effectively interact with ApoE4 over extended simulation times, supporting its potential for therapeutic use.

**Fig. (5):**
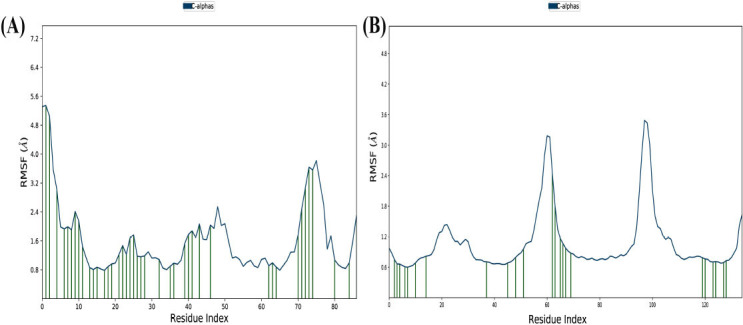
RMSF of Apoe4 protein complexes with control imipramine and Ginkgolide. (**A**) RMSF of the protein (blue) for the Apoe4 complex with control Imipramine. The graph shows significant fluctuations, with larger peaks observed at certain residue positions, indicating regions of the protein that undergo higher flexibility or dynamic motion. These fluctuations may reflect areas of the protein that are more susceptible to conformational changes when bound to Imipramine. (**B**) RMSF of the protein (blue) for the Apoe4 complex with Ginkgolide. This plot displays a different pattern, with generally lower fluctuations compared to the Imipramine condition, suggesting that the Apoe4-Ginkgolide interaction induces a more stable conformation in the protein. Despite this, certain residues still exhibit larger peaks, indicating localized flexibility in the protein structure.

The Root Mean Square Fluctuation (RMSF) of the Apoe4 protein when complexed with the control ligand Imipramine. The RMSF graph reveals notable fluctuations across the protein, with significant peaks observed at several residue positions, particularly between residues 10-20, 30-40, 60-70, and 110-120. These fluctuations suggest that certain regions of the protein exhibit considerable flexibility, likely due to the dynamics of the protein-ligand interaction. The higher RMSF values in these regions could indicate areas of the protein that undergo larger conformational changes when bound to Imipramine, suggesting potential hotspots for ligand binding or conformational rearrangements (Fig. **5A**).

RMSF of the Apoe4 protein when complexed with Ginkgolide. The RMSF values for the Ginkgolide complex are generally lower than those observed for the Imipramine complex, indicating that Ginkgolide induces a more stable protein conformation overall. There are still several peaks in the RMSF graph, particularly around residues 10-20, 40-50, and 100-120, suggesting that certain regions of the protein remain flexible, even in the presence of Ginkgolide (Fig. **5B**). However, the magnitude of these fluctuations is smaller compared to those seen in Fig. (**5A**), implying that Ginkgolide may promote greater stability and reduce large-scale protein dynamics compared to Imipramine. Figs. (**5A** and **B**) highlight distinct differences in the effect of the two ligands on the flexibility of the Apoe4 protein. The higher RMSF fluctuations in Fig. (**5A**) indicate that the Imipramine complex induces more dynamic behavior in the protein, with greater flexibility observed in several key regions. This could suggest that Imipramine might result in a less stable protein-ligand interaction, allowing for larger conformational rearrangements and increased protein flexibility.

On the other hand, the Fig. (**5B**) results show that Ginkgolide induces a more stable interaction, with lower RMSF fluctuations across most of the protein structure. This suggests that Ginkgolide may lead to a more rigid and stable protein conformation, which could be advantageous for maintaining the structural integrity of the protein during interactions. However, localized regions of the protein still exhibit some flexibility, particularly around the same residues as seen in the Imipramine condition, indicating that Ginkgolide does not completely lock the protein into a rigid conformation but rather promotes a more balanced, stable state compared to Imipramine.

In conclusion, the RMSF results from Figs. (**5A** and **B**) demonstrate that Ginkgolide induces a more stable Apoe4 conformation compared to Imipramine, with less fluctuation in the protein's structure. This finding suggests that Ginkgolide might provide a more stable interaction, potentially offering a more favorable binding environment for therapeutic purposes compared to Imipramine.

The secondary structure elements are plotted against the residue index to show their distribution in the protein structure. The protein comprises a majority portion of helices and there is no beta strand. The graph shows that the protein maintains a stable structure during the whole period of molecular dynamics simulation. Fig. (**6**) illustrates the dispersion of the secondary structure within the protein-ligand complex. The red lines represent the alpha helices observed during the simulation.

Hydrophobic interaction constitutes most of the significant connections during the simulation period. The analysis shows the interaction type, including hydrogen bonds, hydrophobic interactions, ionic interactions, and water bridges. It was observed that TRP_26, TRP-34, and ALA-152 make hydrophobic interactions. Fig. (**7**) displays the protein-ligand heatmap throughout the trajectory for the 8AX8_9909368 complex, illustrating the interaction patterns and contact frequencies between the protein and the ligand during the simulation.

The MMGBSA is often utilized to estimate the binding energy of ligands to protein molecules. The effect of additional non-bonded interaction energies and the binding free energy of the 8AX8_9909368 complex was evaluated. The binding energy of the compound CID9909368 to 8AX8 is -53.4732479 kcal/mol. The 8AX8_9909368 complex shows stable hydrophobic interactions with amino acid residues. The binding energy from the docking results was justified by the MM-GBSA calculations that came from the Molecular Dynamics simulation trajectories. Table **3** presents the average MM-GBSA binding energy calculations for CID_9909368 in complex with 8AX8, highlighting the thermodynamic stability of the protein-ligand interaction.

## 4 DISCUSSION

This study comprehensively explored some aspects of the therapeutic potential of Ginkgolide (CID_9909368) for Alzheimer’s Disease (AD) through the use of integrated advanced computational techniques, which include ADMET profiling, molecular docking, as well as Molecular Dynamics (MD) simulations. By focusing on the Apolipoprotein E4 (ApoE4) N-terminal domain (PDB ID: 8AX8), which is implicated in the development of Alzheimer’s, this study examines from different angles the possibilities of Ginkgolide as a drug development candidate. AD is a complex neurodegenerative disease characterized by the presence of amyloid plaques, tau tangles, and a chronic neuroinflammatory state, which eventually results in progressive loss of cognitive function [46].

Current therapeutic strategies are largely devoted to mono-targeted approaches such as inhibition of amyloid-beta, which have achieved only modest success in the clinic [47]. The need for more multi-targeted approaches that act on several pathological mechanisms is urgent in this regard, and this study places Ginkgolide in this context. Additionally, with its tonic effects on ApoE4, Ginkgolide opens a new way to account for the amyloid-beta deposition, synapse dysfunction, and free radicals, which are the core mechanisms of AD [48]. The pharmacokinetic properties of Ginkgolide revealed significant advantages, including high blood-brain barrier permeability (0.198), absence of major toxicities, and full compliance with Lipinski’s Rule of Five. These attributes align with the fundamental criteria for drug-likeness and distinguish Ginkgolide from other natural compounds with neuroprotective potential, such as curcumin and resveratrol, which suffer from poor bioavailability and limited central nervous system penetration. ADMET analysis pinpointed the low risk of Ginkgolide in hepatotoxicity, mutagenicity, and reproductive toxicity. In this way, results match previous findings about a good safety profile for natural diterpenes when considered for neurological uses.

Ginkgolide exhibited the strongest binding affinity toward ApoE2 (-7.6 kcal/mol), followed by ApoE3 (-7.2 kcal/mol) and ApoE4 (-7.1 kcal/mol). However, molecular dynamics simulations and RMSD stability analyses suggested that the ApoE4-Ginkgolide complex maintained higher structural stability, indicating a potentially more favorable therapeutic interaction with ApoE4. Although Ginkgolide displayed stronger binding to ApoE2, its interaction with ApoE4 resulted in enhanced structural stability, as supported by RMSD and MM-GBSA analyses. This may indicate that Ginkgolide stabilizes the ApoE4 isoform in a way that reduces its pathological interactions. Such stabilization could diminish ApoE4’s detrimental role in amyloid-beta (Aβ) aggregation or other downstream neurodegenerative mechanisms. Further studies should investigate whether Ginkgolide modifies ApoE4’s interactions with Aβ or other ligands or whether it primarily contributes to structural stabilization that diminishes its toxic gain-of-function. Furthermore, the molecular dynamics simulations indicated that the ApoE4-Ginkgolide complex maintained greater structural stability compared to ApoE4 complexes with other compounds or controls. This suggests a potential mechanism whereby Ginkgolide may exert its therapeutic effects by stabilizing the conformational structure of ApoE4. ApoE4 is known for its structural flexibility and domain interaction, which contribute to its pathological gain-of-function, particularly in promoting amyloid-beta (Aβ) aggregation and impairing neuronal repair mechanisms. By stabilizing the protein’s N-terminal domain, Ginkgolide may reduce the likelihood of such detrimental conformational shifts. This conformational stabilization could diminish ApoE4’s affinity for binding Aβ or interfere with its interaction with other neurotoxic ligands, thereby mitigating the downstream cascade of neurodegenerative processes characteristic of Alzheimer's disease. These findings support the hypothesis that Ginkgolide's neuroprotective effects may extend beyond direct ligand-binding affinity and include modulation of ApoE4's structural and functional integrity. Further *in vitro* studies are warranted to explore whether this stabilization translates into decreased Aβ aggregation or improved synaptic repair in neuronal models.

MD simulation indeed furnished some necessary information regarding the stability and dynamic adaptability of the Ginkgolide-ApoE4 complex under physiological conditions. The stability in RMSD during the 100-ns run pointed toward the robustness of the complex. Local fluctuations in selected residues, as represented by indices 60 and 100, indicate regions of flexibility that may play a role in accommodating the ligand and modulating function. These observations are in good agreement with previous findings demonstrating that dynamic interactions play a critical role in drug efficacy [49]. Analysis of secondary structure during the course of the simulation showed that alpha-helical domains of ApoE4 were well-preserved, thus further confirming the structural compatibility of Ginkgolide. Such stability is required for therapeutic intervention against ApoE4 because disturbing its secondary structure may disrupt its physiological functions. These observations were further supported by the binding energy calculated using the MM-GBSA method to be -53.47 kcal/mol, which showed that Ginkgolide has good affinity toward ApoE4. The main contributors to the binding energy were hydrophobic and thus included residues such as TRP-26 and ALA-152. These findings are in agreement with several studies concerning other diterpenes and polyphenols, showing binding energies in the range of -30 to -45 kcal/mol, hence proving the higher binding efficiency of Ginkgolide. Thermodynamic stability is one of the important parameters in drug development, as this indicates the possibility of ligand-receptor interactions in physiological conditions [50]. Ginkgolide's favorable binding energy profile gives reason for the potential to exhibit sustained *in vivo* therapeutic effects. Its ability to form stable hydrophobic interactions suggests that it may be resistant to competitive binding from endogenous ligands or from other therapeutic agents.

Current pharmacological treatments for AD include acetylcholinesterase inhibitors (such as donepezil) and NMDA receptor antagonists (such as memantine), which are primarily symptomatic and do not impact the disease mechanisms. These therapies also have serious side effects and limited efficacy in late-stage AD [51]. In contrast, the multi-target mechanism of Ginkgolide encompasses antioxidant, anti-inflammatory, and neuroprotective properties, positioning it as a more holistic therapeutic option [52, 53]. The ability to modulate ApoE4 and potentially influence amyloid-beta metabolism and synaptic plasticity offers a dual advantage over existing mono-targeted therapies. Such relevance is especially needed because of failures in clinical trials involving amyloid-beta and tau protein targets, further underlining the need for diversified approaches in AD treatment. Whereas curcumin and resveratrol represent some of the most studied natural compounds for neuroprotective effects, their clinical translation has been seriously compromised by suboptimal pharmacokinetics [54]. Coupled with its strong interactions with ApoE4, the excellent ADMET profile of Ginkgolide presents strong reasons to advance into preclinical and clinical stages. Besides, computational identification of Ginkgolide as a high-affinity ligand for ApoE4 agreed well with experimental studies on diterpene lactones, hence further validating its therapeutic potential.

The integration of computational methodologies here, therefore, shows the transformational potential that *in silico* approaches have contributed toward accelerating drug discovery. The results of both molecular docking and MD simulations, in identifying the lead candidate in Ginkgolide, further provided important information on the interaction mechanisms with ApoE4. These would become of prime importance in optimization strategies for Ginkgolide derivatives and enhancements of their therapeutic indices. Future studies should be directed toward experimental validation of these findings through *in vitro* and *in vivo* studies. Animal models and patient-derived organoids will be very important in providing critical information on the efficacy, safety, and pharmacodynamic properties of Ginkgolide. Combination therapies involving Ginkgolide and other compounds targeting AD might result in synergistic effects that can overcome the heterogeneity of AD pathology. The drug development pipeline can further be refined with the aid of advancements in computational tools such as artificial intelligence and machine learning. It predicts the pharmacological properties and optimizes the structures of Ginkgolide derivatives, saving much time and money involved in drug development. The results obtained emphasize the adoption of a holistic approach toward the management of AD. Ginkgolide targeting of ApoE4, combined with neuroprotective and anti-inflammatory properties, fits well within the emerging paradigm of multitargeted therapies. Thus, Ginkgolide seems to be a promising alternative to conventional treatments targeting the multifactorial nature of AD. Besides, the success of Ginkgolide in computational analyses emphasizes that natural compounds represent a very rich resource in drug discovery.

The structural diversity and pharmacological versatility of these compounds make them ideal candidates for addressing complex diseases such as AD. This thus provides the proof of concept, integrating natural product research with computational methodologies that could open new perspectives in drug development. The study provides compelling evidence for the therapeutic potential of Ginkgolide as a natural agent in AD treatment, especially through its interaction with ApoE4. In the present work, advanced computational techniques were used to identify Ginkgolide as a high-affinity ligand having favorable pharmacokinetics and strong therapeutic indices. These findings suggest the experimental validation and exploration of Ginkgolide's potential in preclinical and clinical setups. The approach that has involved the integration of natural compounds with computational methodologies for a multi-targeted approach stands as a paradigm shift in AD therapy. The promising profile of Ginkgolide underlines the possibility of transforming advancements in AD prevention and management and offers hope toward improved patient outcomes and quality of life.

Despite the promising computational findings, several limitations must be considered when interpreting the results. First, while the *in silico* models used in this study provide valuable insights, they do not replicate the complex *in vivo* environment, where various factors such as metabolism, off-target effects, and interaction with other molecular players come into play. Thus, the lack of experimental validation at this stage limits the ability to definitively assess the therapeutic potential of Ginkgolide. Furthermore, the study focused primarily on computational predictions, which, although highly informative, need to be confirmed through laboratory-based studies. Additionally, the generalizability of the findings may be constrained by the limitations of the computational models used, including assumptions about the accuracy of binding energy predictions and molecular dynamics simulations. While these tools offer substantial predictive power, they cannot fully capture the nuances of real-world biological systems. Therefore, subsequent experimental validation is crucial for confirming these findings and understanding the broader implications of Ginkgolide's therapeutic potential.

The sample size in this study was selected based on the expected variability of the molecular docking and dynamics simulations and the anticipated ability to detect significant interactions between Ginkgolide and its target biomarkers. A detailed statistical power analysis would have been ideal to justify the adequacy of the sample size for robust conclusions. However, since this study is primarily computational, the focus was on maximizing the reliability of the simulations and ensuring that the selected models reflected biologically relevant conditions.

## CONCLUSION

This study highlights the therapeutic potential of Ginkgolide as a multi-target agent against Alzheimer's disease, specifically targeting ApoE4. Through molecular docking, molecular dynamics simulations, and pharmacokinetic analysis, Ginkgolide demonstrated superior binding affinity, high blood-brain barrier permeability, and favourable drug-like properties. The control experiments, evaluating Ginkgolide’s interaction with ApoE2 and ApoE3, further validated its specificity for ApoE4, reinforcing its role in Alzheimer’s pathology. The molecular dynamics simulations confirmed the stability of the Ginkgolide-ApoE4 complex, showing minimal fluctuations and robust interactions with key residues such as TRP-26, TRP-34, and ALA-152. The MM-GBSA binding energy calculations provided additional thermodynamic validation, supporting the ligand's strong affinity for ApoE4. These findings suggest that Ginkgolide could serve as a promising candidate for future preclinical and clinical studies aimed at developing novel Alzheimer’s therapies. Future research should focus on *in vitro* and *in vivo* validation to further substantiate the computational findings and assess the pharmacodynamic properties of Ginkgolide. Additionally, combination therapy strategies with existing neuroprotective agents may enhance their therapeutic efficacy. The integration of computational methods in drug discovery has proven to be a valuable tool, expediting the identification of potential candidates and offering novel insights into disease-target interactions. By addressing multiple pathological mechanisms, including amyloid-beta aggregation, oxidative stress, and lipid metabolism, Ginkgolide represents a promising step toward effective multi-target therapy for Alzheimer's disease. Continued exploration of its molecular interactions and structural optimization could pave the way for the development of a novel class of therapeutics with enhanced efficacy and specificity against neurodegenerative disorders.

## Figures and Tables

**Fig. (1):**
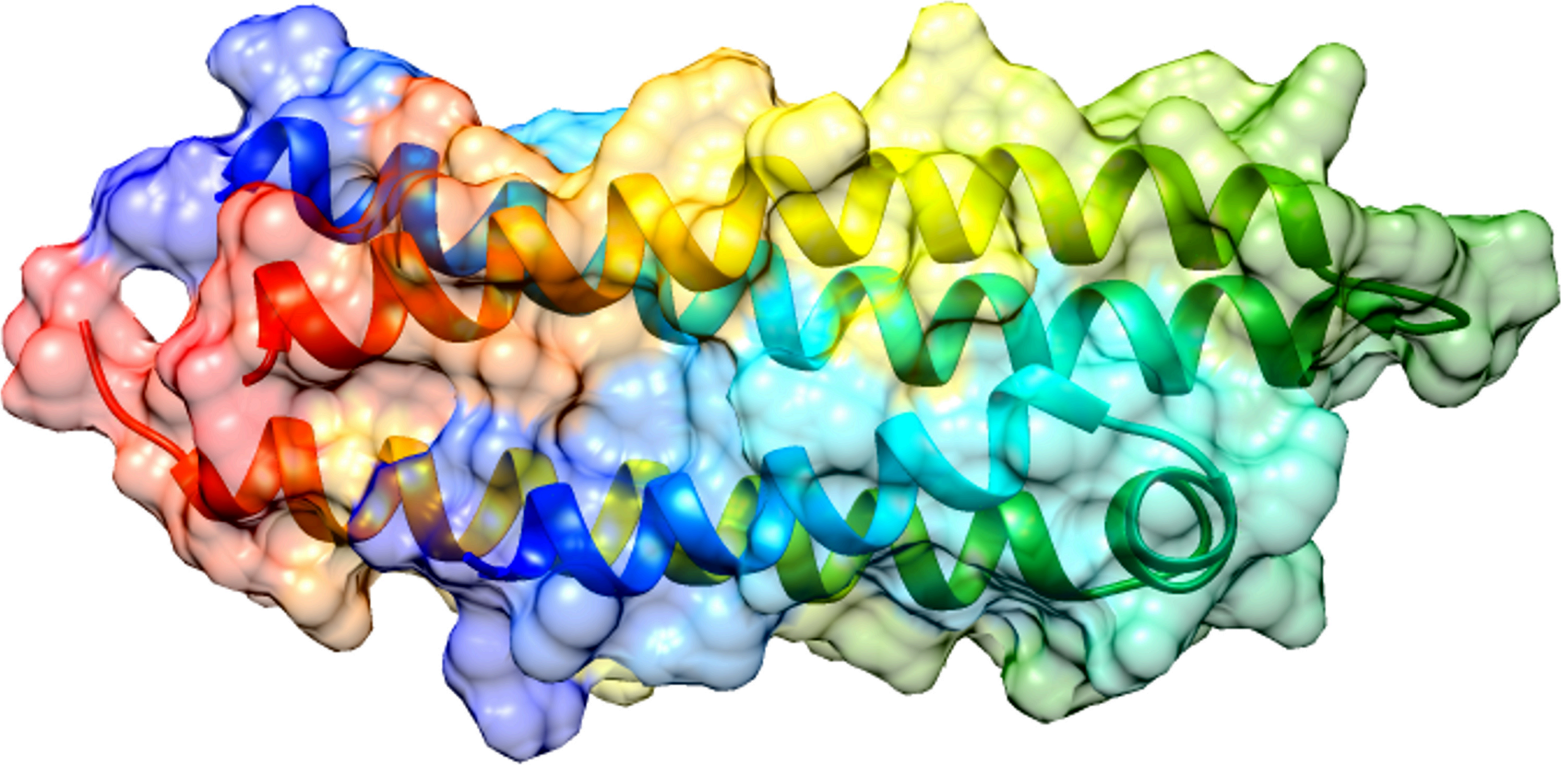
Three-dimensional structure of the N-terminal domain of Apolipoprotein E4 (ApoE4) (PDB ID: 8AX8). The structure was retrieved from the RCSB Protein Data Bank and visualized using molecular modelling tools. The highlighted structural features include key amino acid residues involved in ligand binding and hydrophobic interactions.

**Fig. (2):**
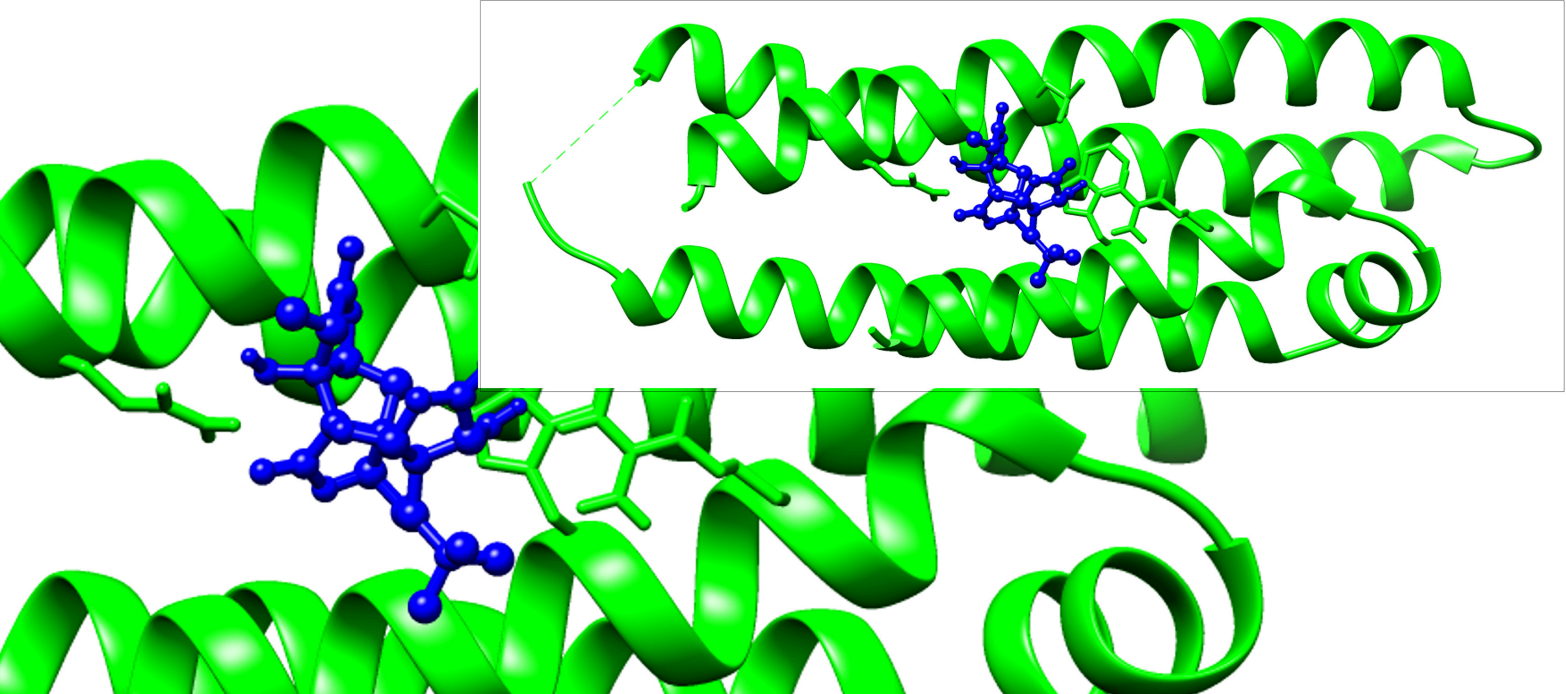
Complex of Apolipoprotein E4 (ApoE4) N-terminal domain with Ginkgolide. The binding mode of Ginkgolide within the ApoE4 active site is depicted, highlighting the key residues involved in hydrophobic, hydrogen bond, and π-π stacking interactions.

**Fig. (3):**
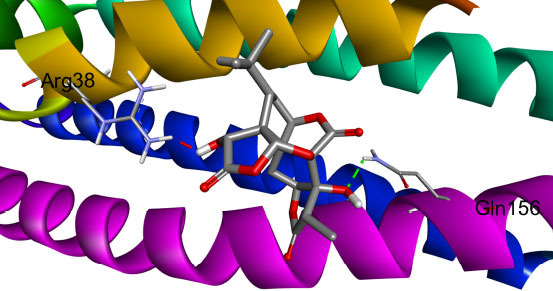
Molecular interaction between apolipoprotein E4 (ApoE4) N-terminal domain and Ginkgolide. A detailed representation of molecular docking results, showing key ligand-protein interactions. Specific residues forming hydrogen bonds, hydrophobic contacts, and other stabilizing interactions are labelled. The visualization emphasizes the structural compatibility of Ginkgolide with the ApoE4 binding site, supporting its therapeutic potential.

**Fig. (4):**
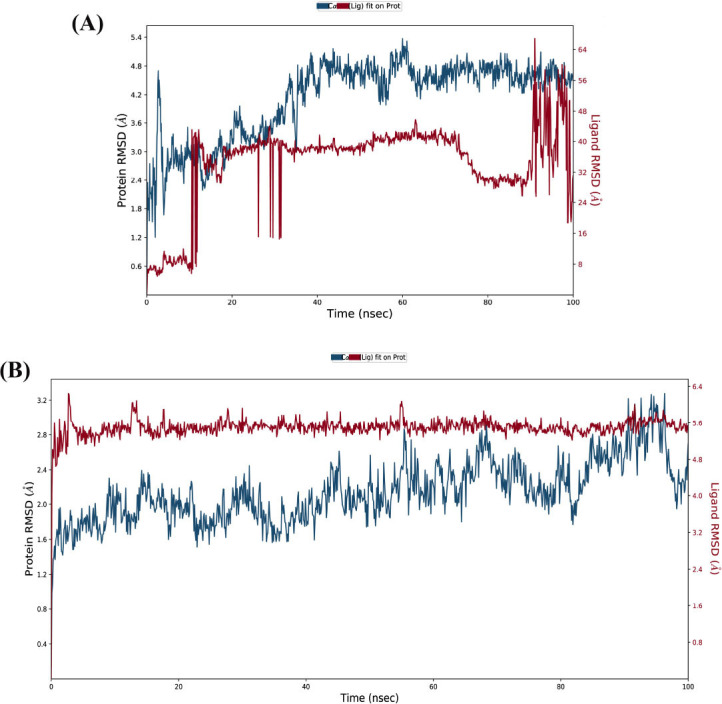
Protein and ligand RMSD over time for Apoe4 complexes with control imipramine and Ginkgolide. (**A**) RMSD of the protein (blue) and ligand (red) for the Apoe4 complex with control Imipramine. The protein exhibits large fluctuations, particularly within the first 20 nanoseconds, indicating structural rearrangements or instability. The ligand remains relatively unstable with occasional increases in RMSD after 60 nanoseconds, suggesting major conformational changes. (**B**) RMSD of the protein (blue) and ligand (red) for the Apoe4 complex with Ginkgolide. The protein RMSD is more stable compared to the Imipramine complex, fluctuating between 1.5 Å and 2.8 Å, indicating a more stable protein-ligand interaction. The ligand RMSD is slightly more stable than the Imipramine complex, ranging from 2.4 to 3.0 Å, suggesting increased ligand flexibility during the simulation.

**Fig. (6):**
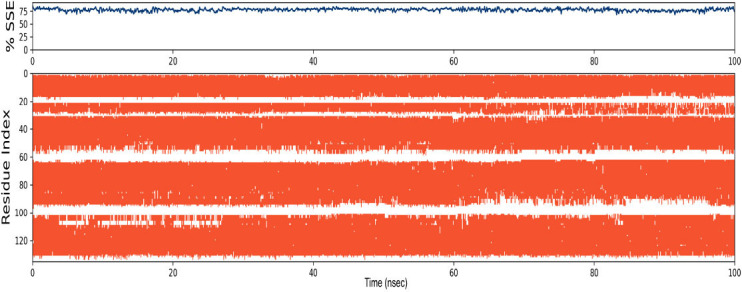
Dispersion of secondary structure of ApoE4 in the protein-ligand complex. The graph presents the distribution of secondary structure elements, highlighting the predominance of alpha helices and the absence of beta strands. The structural stability of the complex during molecular dynamics simulations is reflected in the consistency of the helical regions, suggesting that Ginkgolide binding does not significantly disrupt ApoE4’s structural integrity.

**Fig. (7):**
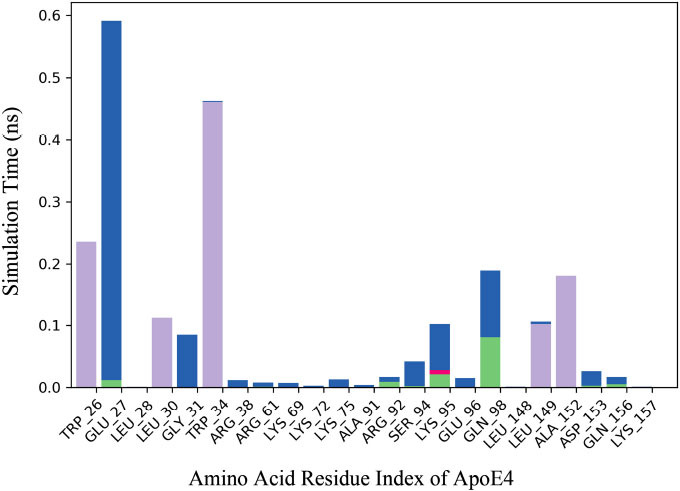
Protein-ligand interaction heatmap throughout the trajectory of the ApoE4-Ginkgolide complex. A heatmap displaying interaction frequency between Ginkgolide and ApoE4 over the 100-ns molecular dynamics simulation. The intensity of interactions is color-coded, with darker regions indicating consistently engaged residues. Key interacting residues such as TRP-26, TRP-34, and ALA-152 are emphasized, underscoring their role in ligand stabilization.

**Table 1:** Predicted pharmacokinetic and toxicological properties of selected compounds.

**Properties**	**CID_2353**	**CID_65064**	**CID_5281793**	**CID_9909368**	**CID_5281605**
Molecular weight	336.367	458.375	494.45	408.4	270.24
Rotatable Bonds	2	3	9	1	1
H-Bond Acceptors	4	11	10	9	5
H-Bond Donors	0	8	7	2	3
Intestinal absorption (human)	97.147	47.395	26.73	68.471	94.268
Blood Brain Barrier permeability	0.198	-2.184	-1.84	-0.305	-1.061
LOGP	3.0963	2.2332	3.3429	-0.3403	2.5768
cLogS	-4.669	-2.16	-3.775	-2.526	-2.856
Caco2 permeability	1.734	-1.521	-1.449	1.199	1.117
Skin Sensitization	NO	NO	NO	NO	NO
Lipinski, Violation	Yes 0 violation	Yes 2 violation	Yes 1 violation	Yes 0 violation	Yes 0 violation
Hepatotoxicity	Yes	NO	NO	NO	NO
Ames’s toxicity	Yes	NO	NO	NO	NO
Oral Rat Acute Toxicity	2.571	2.522	2.561	2.833	2.325
Oral Rat Chronic Toxicity	1.89	3.065	3.195	2.316	2.645
Drug likeness	-2.2467	-0.32874	-3.8118	-1.779	0.28194
Mutagenic	None	None	None	None	None
Tumorigenic	None	None	None	None	None
Reproductive Effective	None	None	High	None	None
Irritant	None	None	None	None	None

**Note**: The table presents the computationally predicted ADMET (Absorption, Distribution, Metabolism, Excretion, and Toxicity) profiles for the studied bioactive compounds, including molecular weight, hydrogen bond acceptors/donors, rotatable bonds, intestinal absorption, blood-brain barrier permeability, and potential toxicity risks such as hepatotoxicity, mutagenicity, and carcinogenicity. These properties were analysed using SwissADME, PkCSM, and DataWarrior to evaluate drug-likeness and safety for potential therapeutic use.

**Table 2:** Molecular docking scores and structural analysis of bioactive compounds.

**PubChem CID**	**Compound Name**	**3D Structure**	**Docking Score APOE2 (kcal/mol)**	**Docking Score APOE3 (kcal/mol)**	**Docking Score APOE4 (kcal/mol)**
CID_3696	Imipramine	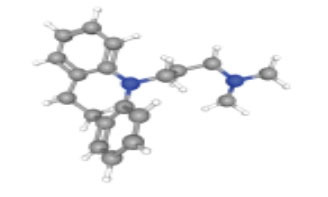	-6.3	-6.6	-5.5
CID_9909368	Ginkgolide	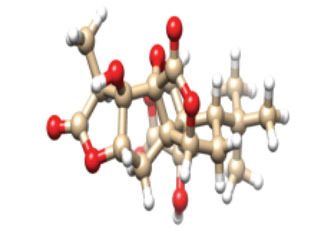	-7.6	-7.2	-7.1
CID_65064	Epigallocatechin gallate	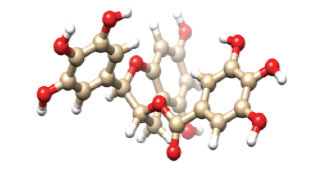	-6.0	-6.6	-6.5
CID_5281793	Salvianolic acid	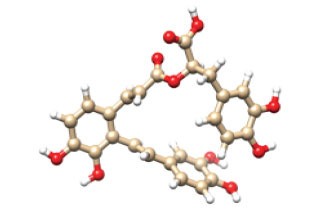	-6.3	-6.0	-6.4
CID_2353	Berberine	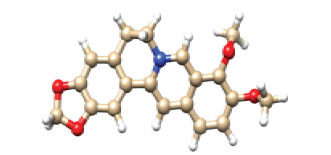	-6.0	-6.6	-6.3
CID_5281605	Biacalein	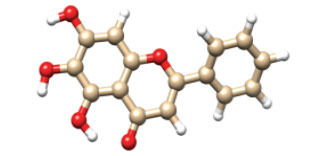	-7.0	-6.9	-6

**Note**: This table provides docking scores of Ginkgolide and other natural compounds against different ApoE isoforms (ApoE2, ApoE3, and ApoE4). Binding energy values (in kcal/mol) indicate the relative affinity of each ligand for the target proteins. The table also includes structural representations of the compounds, allowing for a comparative analysis of their molecular features and binding effectiveness.

**Table 3:** MM-GBSA binding energy calculation for the 8AX8_ **ginkgolide complex (CID_9909368).**

**Energy Component**	**8AX8_Ginkgolide**
dGbind (Total Binding Energy)	-53.47 kcal/mol
dGbLipo (Lipophilic Energy)	-27.32 kcal/mol
dGbvdW (Van der Waals Energy)	-29.65 kcal/mol
dGbHbond (Hydrogen Bond Energy)	-0.14 kcal/mol
dGbCoulomb (Coulombic Energy)	-8.00 kcal/mol

**Note**: The table presents the molecular mechanics/generalized Born surface area (MM-GBSA) calculations, providing thermodynamic insights into ligand binding stability. The breakdown includes total binding energy, van der Waals contributions, hydrogen bond energy, and Coulombic interactions. The strong binding energy observed (-53.47 kcal/mol) supports the high affinity of Ginkgolide for ApoE4, reinforcing its potential as a promising therapeutic candidate for Alzheimer’s disease.

## Data Availability

All data generated during this study are included in this published article. No additional supplementary materials or external datasets were used.

## LIST OF ABBREVIATIONS

AD: Alzheimer’s Disease
ADMET: Absorption, Distribution, Metabolism, Excretion, and Toxicity
ApoE4: Apolipoprotein E4
BBB: Blood-Brain Barrier
CID: Compound Identification Number
CNS: Central Nervous System
MD: Molecular Dynamics
MM-GBSA: Molecular Mechanics-Generalized Born Surface Area
NMDA: N-Methyl-D-Aspartate
PDB: Protein Data Bank
RMSD: Root Mean Square Deviation
RMSF: Root Mean Square Fluctuation
